# Toothbrush Contamination: A Review of the Literature

**DOI:** 10.1155/2012/420630

**Published:** 2012-01-24

**Authors:** Michelle R. Frazelle, Cindy L. Munro

**Affiliations:** ^1^School of Nursing, Virginia Commonwealth University, 1100 East Leigh Street, Richmond, VA 23298-0567, USA; ^2^School of Nursing, University of South Florida, 12901 Bruce B Downs Boulevard, Tampa, FL 33612, USA

## Abstract

Toothbrushes are commonly used in hospital settings and may harbor potentially harmful microorganisms. A peer-reviewed literature review was conducted to evaluate the cumulative state of knowledge related to toothbrush contamination and its possible role in disease transmission. A systematic review was conducted on adult human subjects through three distinct searches. The review resulted in seven experimental and three descriptive studies which identified multiple concepts related to toothbrush contamination to include contamination, methods for decontamination, storage, design, and environmental factors. The selected studies found that toothbrushes of healthy and oral diseased adults become contaminated with pathogenic bacteria from the dental plaque, design, environment, or a combination of factors. There are no studies that specifically examine toothbrush contamination and the role of environmental factors, toothbrush contamination, and vulnerable populations in the hospital setting (e.g., critically ill adults) and toothbrush use in nursing clinical practice.

## 1. Introduction

Toothbrushes play an essential role in oral hygiene and are commonly found in both community and hospital settings. Toothbrushes may play a significant role in disease transmission and increase the risk of infection since they can serve as a reservoir for microorganisms in healthy, oral-diseased and medically ill adults [1]. Contamination is the retention and survival of infectious organisms that occur on animate or inanimate objects. In healthy adults, contamination of toothbrushes occurs early after initial use and increases with repeated use [2, 3]. Toothbrushes can become contaminated from the oral cavity, environment, hands, aerosol contamination, and storage containers. Bacteria which attach to, accumulate, and survive on toothbrushes may be transmitted to the individual causing disease [4, 5]. In the hospital setting, toothbrushes are commonly used for oral care by nurses. There is a need for standardized nursing guidelines to prevent toothbrush contamination, which may increase the risk of infections from potentially pathogenic microorganisms and is clinically relevant for assessing the risks and benefits of oral care and informing nursing practice. This review of peer-reviewed literature was conducted to evaluate the cumulative state of knowledge related to toothbrush contamination, its possible role in disease transmission, and in preparation for a research study related to toothbrush contamination in critically ill adults.

## 2. Methods

A systematic review of the scientific literature was conducted. There were no relevant articles available in print prior to 1977. Articles published from 1977 to 2011, on human subjects and using the English language were obtained. The review included studies that evaluated toothbrush contamination in healthy and oral-diseased adults, guidelines for toothbrush and oral care in both healthy and medically ill persons, hospitalized and nonhospitalized patients, and interventions for reducing contamination of toothbrushes. Experimental and nonexperimental designs were included in the review. The following databases were searched: Pub Med (clinical inquiries and MESH), CINAHL, Cochrane Library, National Guidelines Clearinghouse, Web of Science, and Google Scholar. Key search terms used in the review were *toothbrush, tooth brushing, colonization, bacterial contamination, contamination, oral hygiene, oral health, nursing practice, microbial contamination, *and* adults. *This search strategy was verified by a health sciences librarian. A total of three separate searches were conducted in a systematic fashion using the inclusion and exclusion criteria and search terms. The first search (search 1) identified articles in the selected databases and complete copies of articles that were considered to have met the inclusion criteria were obtained for further review (Table 1). Articles were excluded if they did not meet the inclusion criteria listed above, were conducted on a pediatric population, were duplicates from other databases, or only explored antibacterial methods.

**Figure 1:**
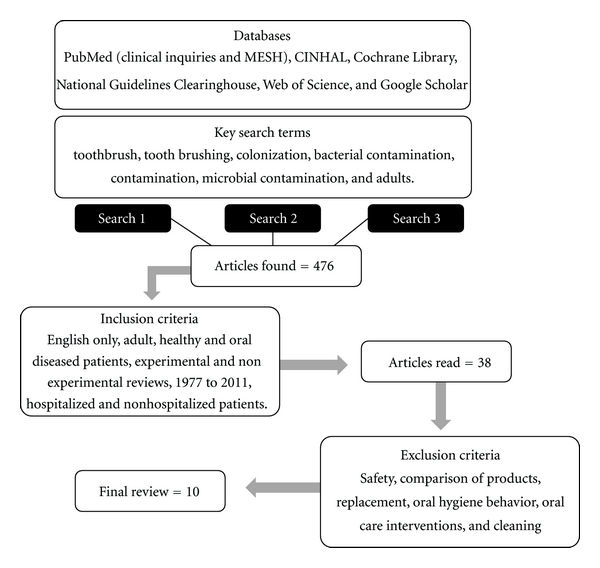
Literature search process.

The second search (search two) included articles identified through cited articles and were reviewed following the same criteria. There were a total of 23 new articles identified through the second search. A third search (search three) was conducted one year after the first search in order to capture any recently published articles. There were three new articles identified in the third search. After a review of the abstracts for the articles obtained through the three searches, a total of 88 relevant articles were identified for further evaluation. After inclusion criteria were applied, 38 articles were selected; after exclusion criteria were applied, ten articles were retrieved to be read in their entirety and included in this review (Figure 1).

## 3. Results

A comprehensive summary of the studies is listed in Table 2. Studies that were reviewed included: seven experimental and three descriptive studies. The selected studies are grouped by setting in vivo, in vitro, and studies that combined both types of settings. The sample sizes ranged from 3 to 103 with the majority of studies having a sample size under 30. Overall, the studies evaluated several perspectives related to toothbrush contamination to include: contamination, methods for decontamination, storage, design, and environmental factors.

### 3.1. Contamination

All of the studies examined toothbrush contamination and found significant bacterial retention and survival on toothbrushes after use [6, 7]. Glass found that toothbrushes from both healthy patients and patients with oral disease contained potentially pathogenic bacteria and viruses such as *Staphylococcus aureus*, *E. coli*, *Pseudomonas,* and herpes simplex virus [1]. Glass also found toothbrushes contaminated with herpes simplex virus 1 in numbers sufficient to cause an infection in the patient [1]. Bunetel et al. found that toothbrushes used by patients with existing oral disease quickly became contaminated [8]. This study also found a significant relationship between repeated use and bacterial retention on toothbrushes and that the oral cavity can be inoculated from a contaminated toothbrush. Several of the studies found that toothbrushes were contaminated before use [5, 9]. Caudry et al. found that toothbrushes are heavily contaminated with normal use [5]. Mehta et al. found that 70% of the toothbrushes in their study became heavily contaminated with pathogenic microorganisms after use [10]. Studies by both Taji and Rogers [11] and Glass [12] found extensive toothbrush contamination after use except in cases where an oral antiseptic, such as mouthwash, was used immediately prior to brushing. Verran and Leahy-Gilmartin found that toothbrushes supported many different bacteria and the amount of growth was varied [13].

### 3.2. Decontamination

Several studies included in this review explored decontamination techniques for contaminated toothbrushes. Bunetel et al. found that toothpaste, mouthwash, and oral antiseptics all decrease microbial load on toothbrushes [8]. Caudry et al. examined toothbrushes in healthy adults as well as possible options for disinfection [5]. Their study found that the toothbrushes became heavily contaminated after use. Soaking the toothbrush in Listerine for 20 minutes prior to and after brushing decreased the microbial load. The use of antimicrobial coated toothbrushes in adults with oral disease was explored by Efstratiou et al. as a means to prevent toothbrush contamination [14]. This study, however, found that coating the bristles with triclosan did not change bacterial growth but the use of toothpaste did. Glass and Jensen explored ultraviolet light as a means of decontamination and found this method to be effective at reducing the bacterial load on toothbrushes [9]. The use of coated tufts and toothpaste was investigated in adult patients with oral disease. Quirynen et al. found that coated tuffs did not inhibit contamination but use of toothpaste did reduce contamination [15]. Mehta et al. found that an overnight immersion in chlorhexidine gluconate was highly effective in decreasing toothbrush contamination and chlorhexidine was more effective than Listerine in reducing the microbial load of bacteria [10]. Sato et al. found that rinsing toothbrushes with tap water resulted in continued high levels of contamination and biofilm [16]. Warren et al. found that the use of regular and triclosan-containing toothpaste resulted in lower toothbrush contamination than no toothpaste use [17].

### 3.3. Storage and Environment

Toothbrushes can become contaminated through contact with the environment, and bacterial survival is affected by toothbrush storage containers. Dayoub et al. found that toothbrushes placed in closed containers and exposure to contaminated surfaces yielded higher bacterial counts than those left open to air [18]. Mehta et al. found that the use of a cap for toothbrush storage increased bacteria survival [10]. Glass found that increased humidity in the environment increased bacterial survival on toothbrushes [12]. In addition, Glass found that bacteria survived more than 24 hours when moisture is present [12].

### 3.4. Design

Toothbrushes are manufactured in a variety of styles. Toothbrush bristles range from soft to hard with different cluster patterns and plastic shapes while toothbrush handles included different plastic shapes and decorative moldings. Different toothbrush design elements were examined by some of the studies. Bunetel et al. found that bacteria become trapped inside the bristles of the toothbrush and bacterial survival is dependent upon the bacteria (aerobic versus anaerobic) and toothbrush design [8]. In addition, the researchers found that solid handles had less bacteria retention and that as the surface area increased, so did the microbial load. Efstratiou et al. found that filament type affected bacterial retention [14]. Toothbrushes with bristles that are frayed and arranged closely together trapped and retained more bacteria [19]. This finding was also echoed in a study by Glass [1] that explored the level of bacterial retention based on toothbrush brand, color and bristle pattern. Contamination was the lowest in soft and round, clear, two bristle row toothbrushes. Glass also found that pathogenic bacteria adhere to plastic after short exposure times [1]. Caudry et al. found that bacteria strongly adhere to the bristles [5]. Mehta et al. found that the retention of moisture and oral debris in the bristles increased bacterial survival [10].

## 4. Conclusions

Due to the limited number of publications specifically related to toothbrush contamination, it was necessary to conduct a preliminary evaluation of the majority of identified articles for this review. For example, several of the articles combined an in vivo examination of bacterial survival on actual patient's toothbrushes and then conducted an in vitro autoinoculation experiment to examine decontamination methods on sterile toothbrushes in the laboratory. This made database searching and identification of articles for the review more challenging. The selected studies all found that toothbrushes of healthy and oral diseased adults become contaminated with potentially pathogenic bacteria from the dental plaque, design, environment, or a combination of factors. The trend identified in the literature is to evaluate methods to reduce toothbrush contamination or toothbrush design rather than evaluating the process related to how the toothbrush initially becomes contaminated, is stored, or is disinfected.

In a vulnerable population such as critically ill adults, pathogenic contamination may increase the risk of infection and mortality. Although some interventions such as chlorhexidine, toothpaste, mouthwash, and ultraviolet sanitizers reduce bacterial survival, oral hygiene practices in the hospital setting by nurses vary. Currently, there are no nursing guidelines related to toothbrush frequency of use, storage, and decontamination. In the hospital setting, the environment as a source of pathogenic bacteria is now a hot topic and the focus of many current infectious disease research studies. Surfaces in close contact with the patient such as bed frames, countertops, sinks, bedside tables, linens, and mattresses may act as fomites. Toothbrushes may come into contact with these surfaces prior to or after use thus increasing risk. While there is significant literature available on environmental contamination and risk for infection, no studies have specifically examined the toothbrush on more vulnerable hospital populations such as critically ill adults.

Toothbrush storage is inconsistent in both community and hospital environments and may increase exposure to pathogenic organisms. The storage conditions of toothbrushes play an important role in bacterial survival: toothbrushes stored in aerated conditions had a lower number of bacteria than those stored in plastic and bacterial growth on the toothbrush increased 70% in a moist, covered environment [10]. In clinical practice, the author has observed that there is no standardized nursing protocol for the storage or replacement of toothbrushes and that some commonly observed nursing practices include storing the toothbrush in the bath basin with other bathing/personal supplies and linens, in a paper towel, in a plastic wrapper, on the bedside table, next to the sink, and in an oral rinse cup at the bedside. These practices may impact the contamination of toothbrushes.

In this review, the majority of studies identified had small sample sizes. Studies with larger sample sizes would be beneficial in future studies. Importantly, despite multiple studies supporting toothbrush contamination and the likely relationship between contamination and disease transmission, there are no studies that specifically examine toothbrush contamination and the role of environmental factors, toothbrush contamination and vulnerable populations in the hospital setting (e.g., critically ill adults), and toothbrush use in nursing clinical practice. Additional descriptive studies to evaluate these relationships would be beneficial and informative for future research. The relationship between environmental factors, toothbrush contamination, and patient oral colonization would inform development of nursing oral care guidelines for adults that minimize risks related to toothbrush contamination.

## Figures and Tables

**Table 1:** Results of Search 1.

Database	Initial number of articles located
PubMed	26
CINAHL	16
Cochrane Library	10
National Guidelines Clearinghouse	None
Web of Science	22
Google Scholar	376

**Table 2:** Studies Selected.

Study	Purpose	Design	Sample	Results
In vitro studies				

Bunetel et al. (2000) [8]	Does retention and survival of microorganisms on toothbrushes pose a threat to patients at risk of infection?	Experimental	*N* = 3 toothbrush types with two series of experiments	Contamination of toothbrushes occurs early in the life of the brush and tends to increase with repeated use.
Dayoub et al. (1977) [18]	To determine the degree of bacterial contamination of toothbrushes after contamination and storage in vented containers or in air.	Experimental	*N* = 103 toothbrushes	The numbers of bacteria on toothbrushes stored in room air after use decrease more quickly than on brushes in containers.
Glass and Jensen (1994) [9]	To evaluate toothbrush design and UV sanitation on microbial growth.	Experimental	*N* = 72 toothbrushes	UV sanitizing kills bacteria; viruses can survive on toothbrushes for 24 hours; toothbrush design, color, opacity, and bristle arrangement are a major factor in retaining microorganisms.

In vivo studies				

Efstratiou et al. (2007) [14]	To examine the contamination and the survival rate of periodontopathic and cariogenic species on new toothbrushes with antibacterial properties after a single use in periodontic patients.	Experimental	*N* = 10 patients; 4 toothbrushes per patient.	Immediately after brushing, the toothbrushes harbored a significant number of microorganisms with no difference between the types of toothbrushes. The antibacterial toothbrush did not limit bacterial contamination.
Mehta et al. (2007) [10]	To determine the extent of bacterial contamination of toothbrushes after use, evaluate the efficacy of chlorhexidine and Listerine in decontamination, and effectiveness of covering the toothbrush head with a cap.	Experimental	*N* = 10 patients	Toothbrushes become contaminated during use; retention of moisture and the presence of organic matter may promote bacterial growth. Toothbrush contamination may lead to colonization and infection. Caps increase bacterial growth. Chlorhexidine was more effective than Listerine.
Quirynen et al. (2003) [15]	To evaluate the effects of coated tuffs and toothpaste on toothbrush contamination.	Experimental	*N* = 8 patients	Toothbrushes become contaminated and toothpaste reduced bacterial growth in toothbrushes.
Taji and Rogers (1998) [11]	To investigate the microbial contamination of toothbrushes.	Descriptive	*N* = 10 patients	Most toothbrushes were contaminated.
Verran and Leahy-Gilmartin (1996) [13]	To evaluate toothbrush contamination using a range of selective and nonselective media.	Descriptive	*N* = 28 toothbrushes	Used toothbrushes supported a wide variety of microorganisms. All media showed growth.

Combination of both in vitro and in vivo studies				

Caudry et al. (1995) [5]	To demonstrate, quantitatively, the presence of microorganisms adherent to toothbrush bristles.	Experimental	*N* = 20 toothbrushes	Toothbrushes, in normal use, are heavily contaminated by microorganisms and the bacteria are extremely adherent to the bristles.
Glass and Lare (1986) [6]	Do toothbrushes harbor pathogenic microorganisms and if there is a correlation between contaminated brushes and the presence of disease.	Descriptive	*N* = 30 toothbrushes	Toothbrushes can harbor pathogenic microorganisms.
